# Identification of key gene networks controlling vernalization development characteristics of *Isatis indigotica* by full-length transcriptomes and gene expression profiles

**DOI:** 10.1007/s12298-021-01110-2

**Published:** 2021-12-24

**Authors:** Pan Wang, Dong Liu, Fu-Hong Yang, Hui Ge, Xin Zhao, Hong-Gang Chen, Tao Du

**Affiliations:** 1 Gansu University of Chinese Medicine, Lanzhou, 730000 China; 2 Pingliang Academy of Agricultural Sciences, Pingliang, 744000 China

**Keywords:** *Isatis indigotica* Fort*.*, Vernalization, Nanopore sequencing, Transcript, WGCNA

## Abstract

*Isatis indigotica* Fort*.*, as a common Chinese medicinal raw material, will lose its medicinal value if it blooms early, so it is highly valuable to clarify the induction mechanism of the vernalization of *I. indigotica* at low temperature. In this study, the concentrations of soluble sugar, proline, glutathione and zeatin in two germplasms of *I. indigotica* with different degrees of low temperature tolerance (Y1 and Y2) were determined at 10 days, 20 days and 30 days of low-temperature treatment, and the full-length transcriptome of 24 samples was sequenced by Nanopore sequencing with Oxford Nanopore Technologies (ONT). After that, the data of transcripts involved in the vernalization of *I. indigotica* at low temperature were obtained, and these transcripts were identified using weighted gene co-expression network analysis (WGCNA). The results revealed the massive accumulation of soluble sugar and proline in Y1 and Y2 after low temperature induction. A total of 18,385 new transcripts, 6168 transcription factors and 470 lncRNAs were obtained. Differential expression analysis showed that gibberellin, flavonoids, fatty acids and some processes related to low temperature response were significantly enriched. Eight key transcripts were identified by WGCNA, among which *ONT.14640.1, ONT.9119.1, ONT.13080.2* and *ONT.16007.1* encodes a flavonoid transporter, 9-cis-epoxycarotenoid dioxygenase 3 (NCED3), growth factor gene and L-aspartate oxidase in plants, respectively*.* It indicated that secondary metabolites such as hormones and flavonoids play an important role in the vernalization of *I. indigotica*. qRT-PCR proved the reliability of transcriptome results. These results provide important insights on the low-temperature vernalization of *I. indigotica*, and provide a research basis for analyzing the vernalization mechanism of *I. indigotica*.

**Supplementary Information:**

The online version contains supplementary material available at 10.1007/s12298-021-01110-2.

## Introduction

*Isatis indigotica* Fort*.,* a biennial herbaceous plant belonging to the *Cruciferae* family and classified into *Isatis*, has high medicinal value. Its dried roots Ban Lan Gen (*Radix Isatidis*) and dried leaves Da Qing Ye (*Folium Isatidis*) have good efficacies such as heat-clearing and detoxification, blood cooling and macula removal, and sore throat relieving (Chinese Pharmacopoeia Commission 2020). *Radix Isatidis* is one of the most commonly used Chinese herbal medicines. Modern pharmacological studies have revealed that *Radix Isatidis* contains many antiviral constituents represented by (R)- and (S)-Goitrin (Lin et al. 2005; Hsuan et al. 2009; Chen et al. 2021), and *Folium Isatidis* mainly contains chemical constituents such as indigo and indirubin (Wu et al. 2011; Jie et al. 2017; Chang et al. 2012). During the current outbreak of COVID-19 around the world, *Radix Isatidis* and Chinese patent medicines with *Radix Isatidis* as raw materials have played vital roles in the prevention and treatment processes (Ma et al. 2021; An et al. 2021a, b; Xu and Zhang 2020). *I. indigotica* was widely planted in Italy and other European regions in the twelfth to seventeenth century, and it was regarded as a vital type of dye plant, from which indigo could be extracted (Garcia-Macias and John 2004; Salvini et al. 2008; Spataro et al. 2007). At present, the emphasis of research on *I. indigotica* has been mainly laid on its chemical constituents (Zang et al. 2020; Zhang et al. 2020), pharmacological activities (Liang et al. 2020; Lotts et al. 2020; Luo et al. 2019) and clinical applications (Wang et al. 2020; Zhang et al. 2016a, b), including on the biosynthetic pathway of indole alkaloids (Chen et al. 2013; Zhang et al. 2016a, b). However, as a biennial plant, it is easily subjected to bolting and flowering after vernalization at low temperature, which induces lignification in roots, thereby leading to the loss of its medicinal value (Chinese Pharmacopoeia Commission 2020; Zhao et al. 2016). Besides, different sowing time in different areas and the abnormal climate change in recent years have dramatically affected the yield and quality of *I. indigotica (*Zhao et al. 2017). Therefore, it is increasingly important to clarify the important mechanisms by which *I. indigotica* senses low temperature and regulates bolting and flowering, and to search for key functional genes or pathways. At present, the vernalization mechanism of *I. indigotica* remains elusive, and the research on its genomes, transcriptomes and low-temperature stress still has limitations (Kang et al. 2020; Qu et al. 2021; Tang et al. 2014). In addition, *A. thaliana (*Arabia et al. 2021; Hatano-Iwasaki and Ogawa 2012; Lei et al. 2019), radish (Nie et al. 2016a, b; Nie et al. 2016a, b) and other plants belonging to the *Cruciferae* family are the subjects of current studies on the gene functions related to the response to low temperature and early bolting and flowering.

As the high-throughput sequencing technology advances, transcriptome sequencing has been widely applied to explore the expression of functional genes at the genomic level and identify key genes involved in the biological processes. In recent years, constant progress has been observed in the transcriptome sequencing technology. With solutions to the short sequencing fragments of the second-generation transcriptome sequencing technology, the emerging third-generation sequencing technology has been successfully applied in the functional genomics research of maize (Wang et al. 2016; Zhang et al. 2021), loquat (Pan et al. 2020), poplar (Chao et al. 2019; Luo et al. 2021), etc. *G*ene family and stress-tolerant genes have been found using the second-generation sequencing technology (Ma et al. 2017; Qu et al. 2019; Qin et al. 2020), which show defects in gene function annotation and pathway analysis. Relative to the second-generation sequencing technology, the third-generation sequencing technology can achieve the processing of large amounts of data and the reading of long sequences and full-length (FL) gene transcripts, and it is more accurate in gene function annotation (Eid et al. 2009).

Weighted Gene Co-Expression Network Analysis (WGCNA) takes advantage of the interrelated characteristics of various life activities in plants to obtain many expression characteristics of life activities using high-throughput sequencing technology and calculates the correlation between genes to construct the scale-free network topology, that is, it links most genes with regulatory relationships with key regulatory genes as the core (Zhang and Horvath 2005). In most cases, this method is applied to identify the biological relationship between genes related to plant traits, and it also plays a role in the identification of key regulatory genes under the low-temperature induction in plants. Using WGCNA, it has been verified that ethylene, jasmonic acid, zinc finger-containing transcription factors (TFs) and auxin are involved during vernalization in *A. thaliana* acclimated at low temperature (Li et al. 2021). Li et al. (2020) identified that a gene *CpFT1* acclimated at low temperature in *Chimonanthus praecox* using WGCNA, and found that its overexpression in *A. thaliana* can promote flowering.

In this study, the FL transcriptomes of *I. indigotica* seedlings with different vernalization characteristics under low temperature stress were obtained by Nanopore single molecule real-time sequencing technology. Additionally, the transcript regulatory networks of soluble sugar, glutathione, proline and zeatin affected by the vernalization of *I. indigotica* were identified by WGCNA, which will be beneficial for the identification of functional genes associated with the responses of *I. indigotica* to low temperature stress and the analysis of their molecular mechanisms.

## Materials and methods

### Plant materials and treatments

The seedlings of Y1 and Y2 growing till two leaves and a bud stage in the seedling cup were used as experimental materials, and the growth conditions was set as far as uniform. Then the seedlings were treated in a constant temperature incubator at 4 °C and exposed to light and darkness for 12 h each day, light intensity 2000lx, followed by sampling at 0 days (control), 10 days, 20 days and 30 days, respectively under the same relative humidity and light, which were marked as T1, T2, T3 and T4. During sampling, all the seedlings were washed and sucked dry, immediately after which they were frozen in liquid nitrogen and stored at − 80 °C. A total of 24 samples (Y1 and Y2) received the same treatments, with three biological replicates in each treatment.

### Detection of physiological indexes induced by low temperature

The test kits for soluble sugar (Batch No.: 20201124), glutathione (Batch No.: 20201115) and proline (Batch No.: 20201115) were provided by Nanjing Jiancheng Bioengineering Institute. The test kit for zeatin (Batch No.: 202011) was purchased from Shanghai Qincheng Biotechnology Co., Ltd. A total of 1 g of soluble sugar was extracted and ground to be prepared into homogenate by adding 9 mL of distilled water. Then the homogenate was poured into a covered centrifuge tube and kept in boiling water for 10 min. After cooling, the homogenate was centrifuged at 4000 rpm for 10 min at normal temperature, and the supernatant was diluted with distilled water for 10 times and shaken evenly to prepare sample supernatant for later use. Subsequently, 1 g of glutathione was extracted and ground into tissue homogenate by adding 9 mL of normal saline, and the homogenate was centrifuged at 2500 rpm for 10 min, after which the supernatant was taken to be measured. Moreover, 1 g of proline was extracted and added with 9 mL of reagent I to be prepared into tissue homogenate through mechanical homogenization in an ice-water bath. Then the homogenate was centrifuged at 3500 rpm for 10 min, and the supernatant was taken for later measurement. Additionally, 1 g of zeatin was extracted, added with 9 mL of PBS and ground into tissue homogenate. After that, the homogenate was centrifuged at 1000 rpm for 20 min, and the supernatant was taken for subsequent measurement.

### RNA extraction, library construction, and sequencing

1 μg of total RNA was prepared for complementary deoxyribonucleic acid (cDNA) library construction using cDNA-PCR sequencing kit (SQK-PCS109) provided by Oxford Nanopore Technologies Inc. (ONT). A cDNA library was added to FLO-MIN109 Flowcell and run on the PromethION platform provided by Biomarker Technologies Inc. (Beijing, China). Firstly, the original reads were filtered, with the minimum average read quality score = 6 and the minimum read length = 200 bp. Secondly, the FL non-chemical (FLNC) transcripts were determined by searching for primers at both ends of the reads.

### Transcriptome analysis and identification of differentially expressed transcripts (DETs)

Mimimap2 (Heng 2018) software was utilized for comparison with the reference genome (Kang et al. 2020) to obtain FLNC transcripts, and then the FL sequences obtained in the previous step were processed to obtain consistent sequences. Later, the expression level was quantified in StringTie (Pertea et al. 2015), and the differential expressions among sample groups were evaluated using DESeq2 (Love et al. 2014).

### Functional enrichment, TF prediction, and SSR analysis

The new transcript sequences obtained were compared with NR, Swissprot, GO, COG, KOG, Pfam and KEGG databases to obtain annotation information of transcripts. GO enrichment analysis of DETs, statistical enrichment of KEGG pathway, TF identification and identification of SSR of transcriptomes were carried out using R package GOseq (Young et al. 2010), KOBAS software (Xie et al. 2011), iTAK (Zheng et al. 2016), and MISA (Beier et al. 2017), respectively.

### Construction and module identification of co-expression network

The transcript expression matrix of 24 samples were used to filter the genes with CPM value < 1. Besides, R software (R version 3.4.4) and WGCNA (R version 1.6.6) package were employed to construct weighted gene co-expression network and divide related modules (Langfelder and Horvath 2008). Meanwhile, the soft threshold was calculated using pick Soft threshold in WGCNA package, and the optimum power value for the optimal network construction was selected. Furthermore, a scale-free network was constructed using blockwise Modules, with default parameter values. Finally, the visualized analysis of the expression network was performed using Cytoscape (Su et al. 2014).

### qRT-PCR analysis

Transcripts of 6 genes were selected for qRT-PCR validation. Primer 3 was used to design quantitative PCR primers, and M-MuLV first-strand cDNA synthesis kits (TaKaRa) were employed to purify the extracted RNAs and reversely transcribed them into cDNAs. Then the fluorescence quantitative analysis was carried out by 2 × SG Rapid qPCR Mix kit (TaKaRa). The primers used are listed in Supplementary Table S1. The reaction volume of qRT-PCR amplification was 15 μL, including 7.5 μL of 2 × SG Rapid qPCR Master Mix, 0.6 μL of each primer, 40 ng cDNA templates and 3.3 μL of ddH_2_O. PCR conditions are as follows: (denaturation at 95 °C for 30 s, denaturation at 95 °C for 5 s and annealing/extension at 60 °C for 30 s) × 40 cycles. Ultimately, with actin as an internal control gene, 2^−ΔΔCT^ method was adopted to calculate the expression of related genes, which was conducted for 3 times.

## Results

### Low temperature-induced physicochemical changes in endogenous metabolism of *I. indigotica*

**Fig. 1:**
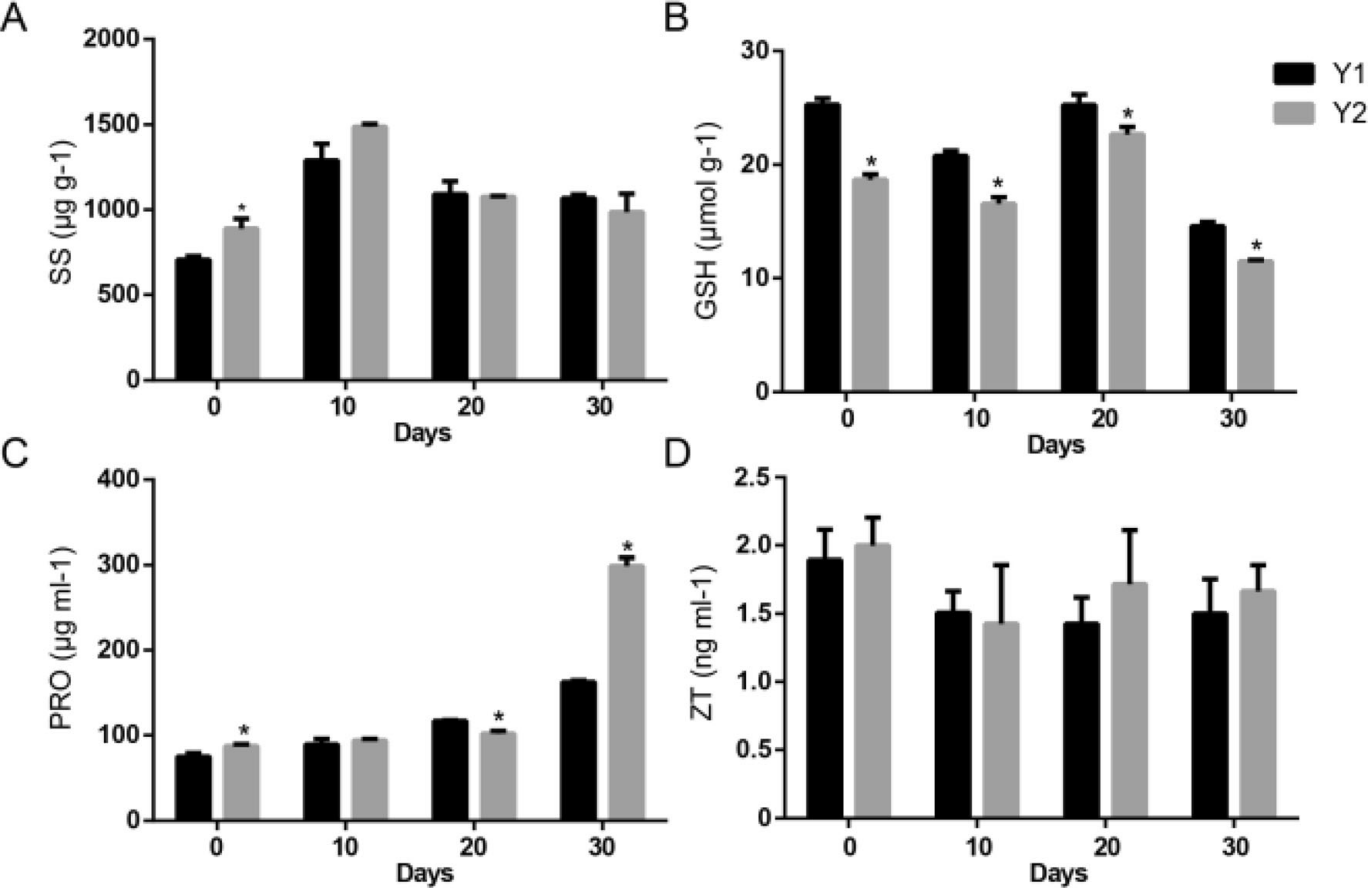
Determination of physiological indexes in Y1 and Y2 under low-temperature induction for 30 days. Determination of soluble sugar (**A**), glutathione (**B**), proline (**C**) and zeatin (**D**) concentration under low-temperature induction for 30 days. The x-axis represents the days of low-temperature induction, and the y-axis represents the concentration of the measured index

The concentrations of soluble sugar, proline, glutathione and zeatin in the seedlings of two germplasms of *I. indigotica,* namely, Y1 and Y2 (both are insensitive to low temperature; Y1 has a higher bolting rate and flowers significantly earlier than Y2), at low temperature were determined at 0 days, 10 days, 20 days and 30 days, respectively. It was discovered that soluble sugar accumulated in Y1 and Y2 at different stages of low-temperature induction. The concentration of soluble sugar was the highest at 10 days after low-temperature induction, and it was significantly higher in Y2 than that in Y1 (Fig. 1A). After measurement, it was found that compared with that in control, the concentration of glutathione in *I. indigotica* after low-temperature induction was decreased and it increased at 20 days after induction, and it was remarkably higher in Y1 than that in Y2 at each stage (Fig. 1B). Moreover, the concentration of proline displayed no obvious differences in *I. indigotica* between control and at 10 days and 20 days after induction, and it dramatically rose at 30 days after induction. Besides, this rise was sharp in Y2 (Fig. 1C). Furthermore, detection also revealed that the concentration of zeatin in Y1 and Y2 declined to a certain extent (Fig. 1D), but it was higher in Y2 than that in Y1 at 20 days after induction.

### Nanopore sequencing statistics of FL transcriptomes of *I. indigotica* under low-temperature treatment

The transcriptome data of Y1 and Y2 at low temperature were compared by Nanopore FL sequencing. There were 24 samples in total, with three biological replicates in each group (Table 1), generating 41.24 million clean reads, and finally 34,329 transcript sequences were obtained. And the FL reads between 86.17 and 88.39% were obtained. The comparison with reference genomes showed that the mapping rate ranged from 96.82 to 97.83%, and 18,385 new transcripts were identified, which enriched the available genome information of *I. indigotica*.

**Table 1:** FL sequence data statistics

Sample ID	Number of clean reads	Number of FL reads	FL percentage (FL%)
Y1T1-1	1,602,599	1,391,641	86.84
Y1T1-2	1,483,412	1,298,111	87.51
Y1T1-3	1,537,791	1,330,526	86.52
Y1T2-1	2,580,298	2,238,140	86.74
Y1T2-2	1,820,551	1,582,151	86.91
Y1T2-3	1,804,455	1,574,278	87.24
Y1T3-1	1,631,957	1,440,318	88.26
Y1T3-2	1,680,488	1,455,894	86.64
Y1T3-3	1,688,362	1,474,633	87.34
Y1T4-1	1,661,199	1,459,456	87.86
Y1T4-2	1,699,129	1,501,824	88.39
Y1T4-3	1,788,443	1,580,108	88.35
Y2T1-1	1,660,251	1,447,775	87.20
Y2T1-2	1,561,540	1,353,875	86.70
Y2T1-3	1,924,762	1,658,574	86.17
Y2T2-1	1,738,488	1,504,980	86.57
Y2T2-2	1,769,897	1,547,472	87.43
Y2T2-3	1,632,278	1,424,839	87.29
Y2T3-1	1,699,253	1,508,608	88.78
Y2T3-2	1,756,976	1,530,234	87.09
Y2T3-3	1,659,921	1,461,467	88.04
Y2T4-1	1,635,174	1,433,631	87.67
Y2T4-2	1,569,343	1,378,388	87.83
Y2T4-3	1,657,924	1,453,889	87.69

**Fig. 2:**
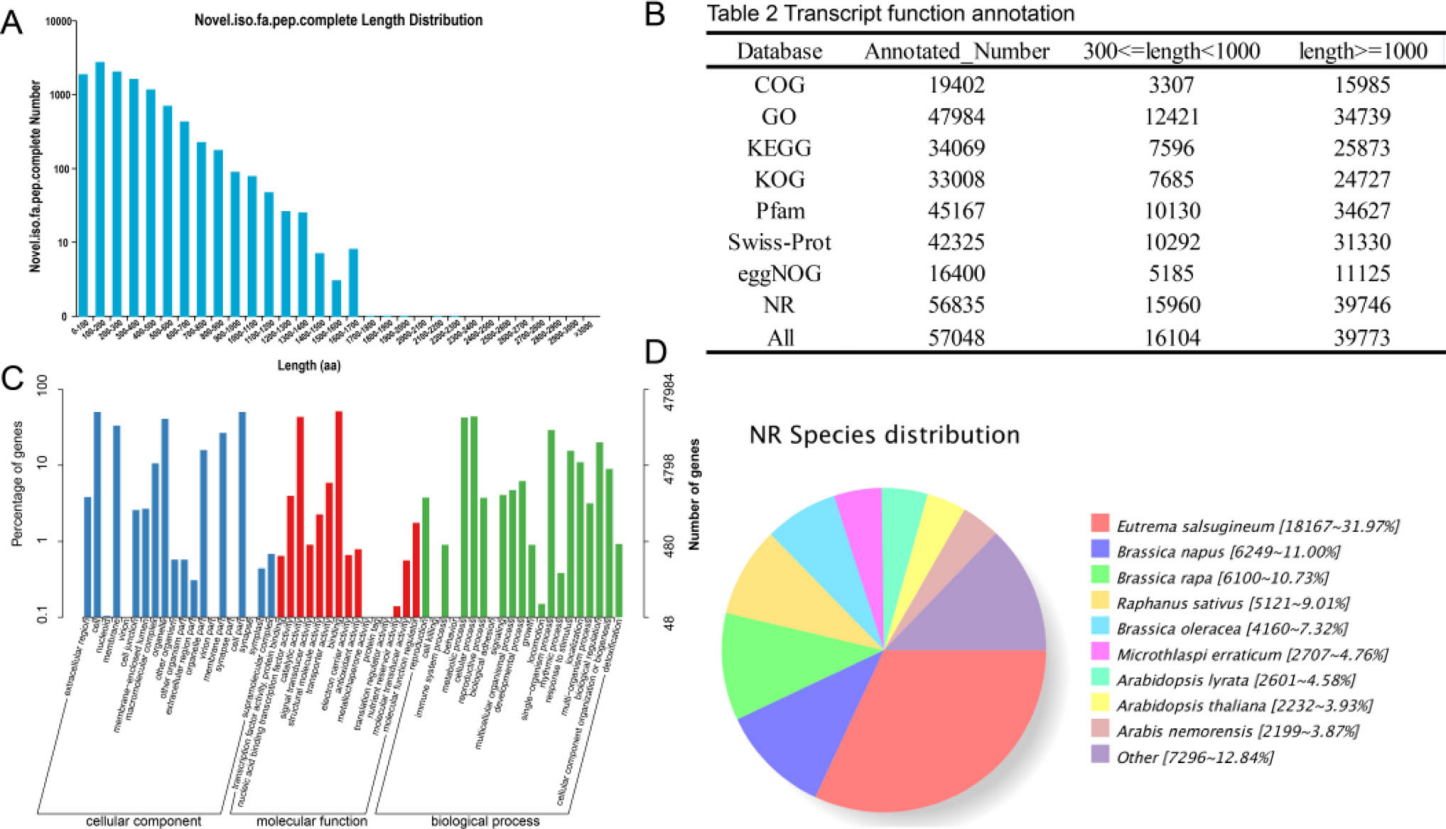
Statistics of *I. indigotica* transcriptome sequencing results under Nanopore low-temperature stress. **A** CDS length distribution in the whole ORF. The x-axis represents the coding sequence length, and the y-axis represents the predicted number of ORFs. **B** Statistics of annotation numbers of ORF sequences with different assembly lengths in COG, GO, KEGG, KOG, Pfam, Swiss-Prot, egg-NOG and NR databases. **C** GO function annotations of identified transcripts. The x-axis indicates the type of GO annotations, y-axis on the left indicates the percentage of transcripts in the total number of genes belonging to this GO function, and the y-axis on the right indicates the number of transcripts. **D** Functional annotations of the identified transcripts based on NR database

Subsequently, the coding region and amino acid sequence of the new transcripts obtained were predicted, and a total of 17,165 open reading frames (ORFs) were obtained, including 11,169 complete coding sequences (CDSs), whose length distribution is shown in Fig. 2A. Next, the identified transcripts were subjected to functional annotation based on clusters of Orthologous Groups (COGs), Gene Ontology (GO), Kyoto Encyclopedia of Genes and Genomes (KEGG), Clusters of Eukaryotic Orthologous Groups (KOGs), protein families (Pfam), Swiss protein (Swiss-Prot), evolutionary genealogy of genes: Non-Supervised Orthologous Groups (egg-NOG) and Non-Redundant Protein Sequence (NR) databases (Fig. 2B). After that, a total of 57,048 annotations were obtained, among which the largest number of annotations appeared based on GO database (47,984) and 34,069 annotations were obtained based on KEGG database. Sequences with a length of 300–1000 bp had 16,104 annotations, and those with a length of more than 1000 bp had 39,773 annotations. According to the statistics of GO annotations (Fig. 2C), the largest number of annotations appeared in "integral component of membrane" (11,499, GO: 0016021) among cellular components, in "ATP binding" (5788, GO: 0005524) among molecular function, and in "cellular processes" (2716, GO: 0009987) in cellular processes. The function of *I. indigotica* transcripts was annotated by NR database (Fig. 2D), among which the largest number of annotations was 18,167 in *Eutrema salsugineum*, accounting for about 37.97%, and there were 6249 annotations in *Brassica napus*, accounting for about 11%.

### TF, simple sequence repeat (SSR) and long non-coding ribonucleic acid (lncRNA) annotation statistics of *I. indigotica* under low-temperature treatment

**Fig. 3:**
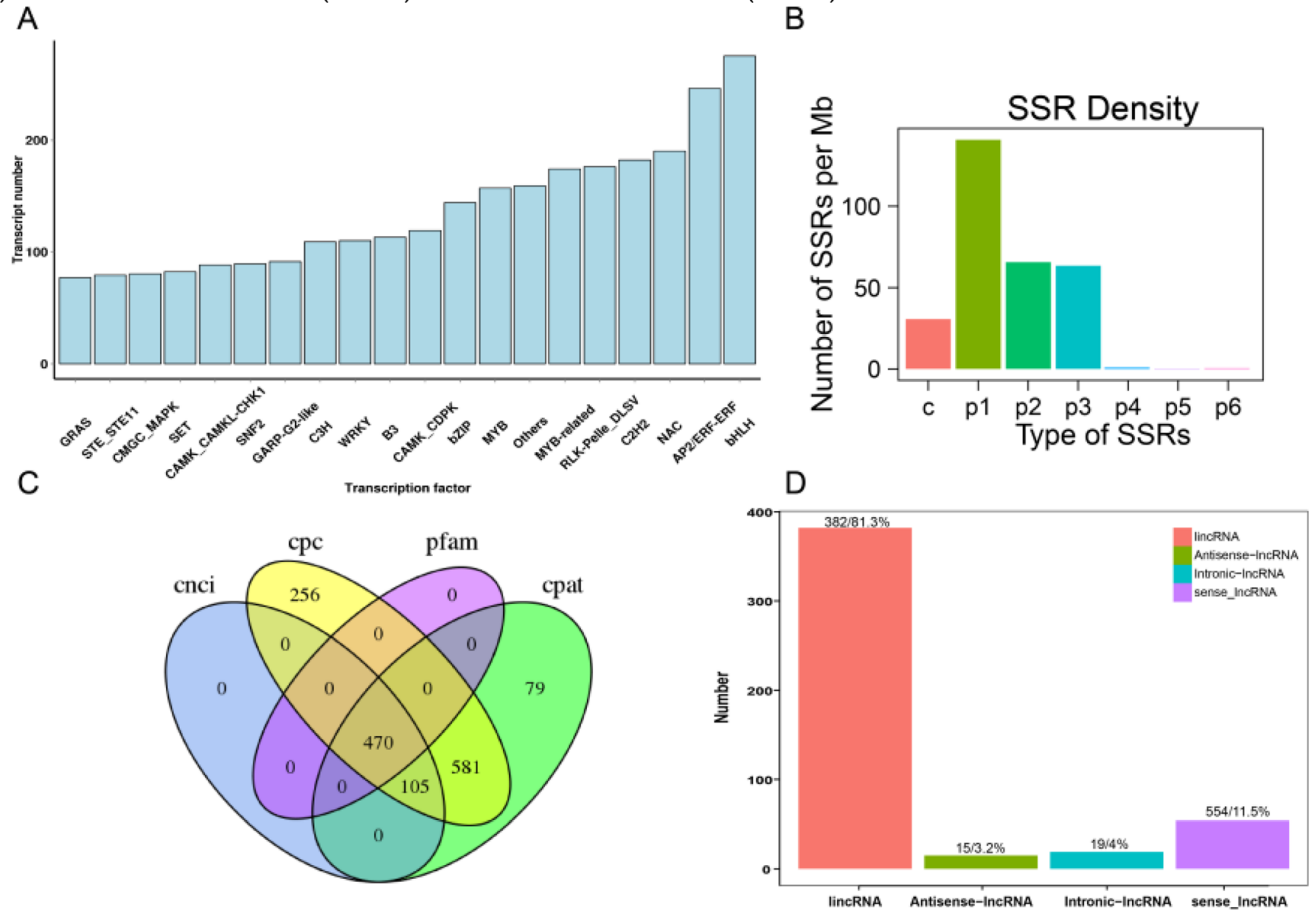
TFs, SSRs and lncRNAs identified by sequencing *I. indigotica* transcriptome after low-temperature stress. **A** Statistics of the number of TFs in transcripts identified. The x-axis indicates the type of TFs, and the y-axis indicates the number of TFs annotated to the type. **B** Statistics of the number of different types of SSRs in transcripts identified. The x-axis reflects the SSR type. Specifically, c:at least two SSRs with the distance of less than 100 bp, p1: mononucleotides repeated at least 10 times, p2: dinucleotides repeated at least 6 times, p3: trinucleotides repeated at least 5 times, p4: tetranucleotides repeated 5 times, p5: pentanucleotides repeated at least 5 times, p6: hexanucleotides repeated at least 5 times. The y-axis reflects the number of transcripts of these SSR types. **C** Venn diagram of lncRNAs identified using CNCI, CPC, Pfam and CPAT methods. **D** LncRNA types classified according to the position of the lncRNAs on the reference genome. The x-axis displays lincRNA, antisense-lncRNA, intronic-lncRNA and sense-lncRNA. The y-axis indicates the number annotated to these types

The candidate TFs were predicted from the identified transcripts (Fig. 3A), and 6168 candidate TFs were obtained, among which there were 275 bHLH TFs and 272 AP2/ERF TFs. As shown in Fig. 3B, the number of SSRs in transcripts, mainly including mononucleotides, dinucleotides, trinucleotides, tetranucleotides, pentanucleotides, hexanucleotides and mixed types (i.e., containing at least two SSRs, with a distance of less than 100 bp), were determined, and a total of 30,952 SSRs were finally obtained, including 15,918 SSRs of mononucleotide type and 51 SSRs of hexanucleotide type.

Later, transcripts were identified by Coding Potential Calculator (CPC), Coding-Non-Coding Index (CNCI), Coding-Potential Assessment Tool (CPAT) and Pfam database (Fig. 3C). These tools identified 1412, 575, 1235 and 470 transcripts, respectively, and a total of 470 lncRNAs were identified. According to the annotation information of lncRNAs in reference genomes, the 470 lncRNAs were classified into four categories (Fig. 3D), namely, long intergenic non-coding RNAs (lincRNAs) (n = 382, 81.3%), antisense-lncRNAs (n = 15), Intronic-lncRNAs (n = 19) and sense-lncRNAs (n = 54).

### Identification and functional analysis of differential transcripts under low-temperature stress

**Fig. 4:**
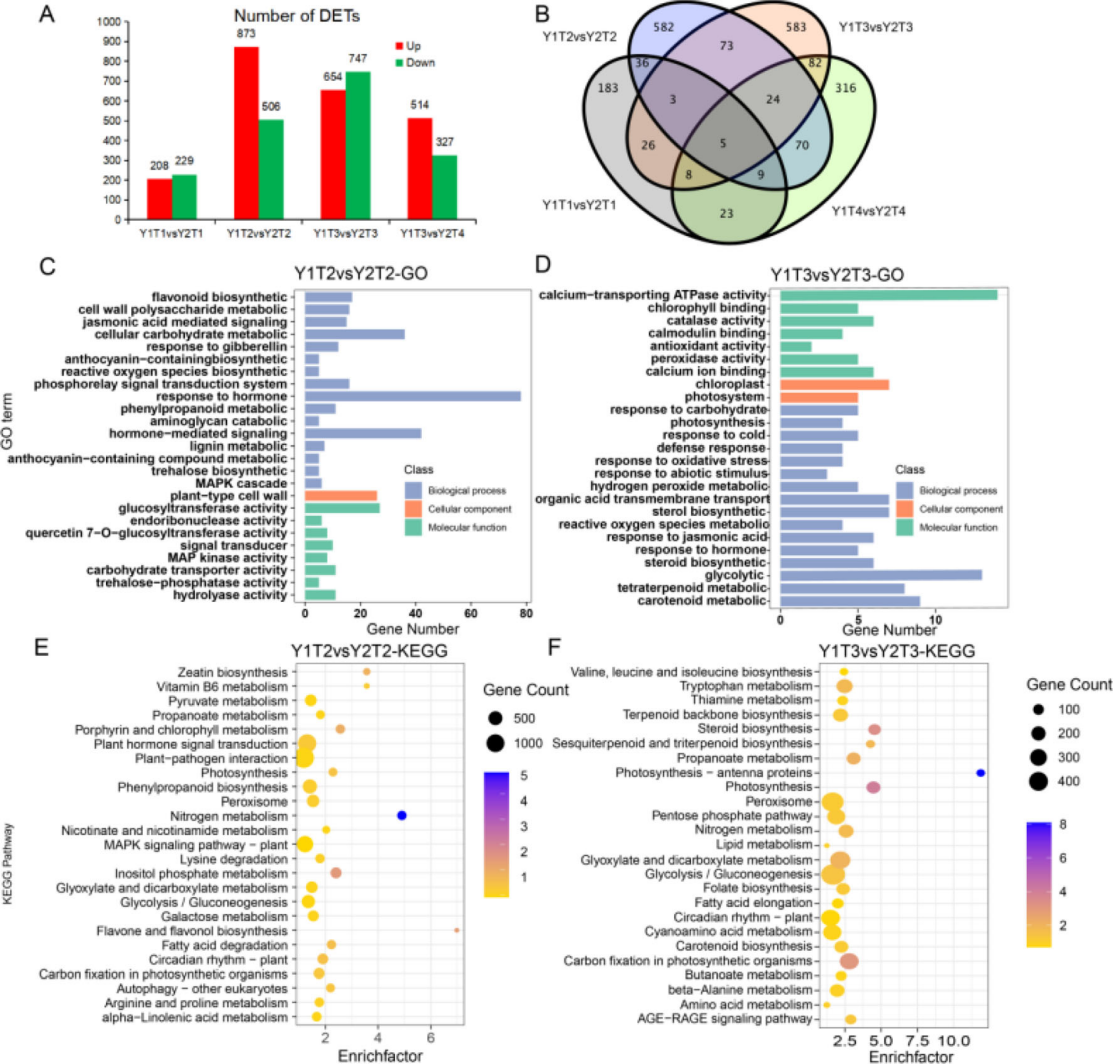
**A** Identification of the number of differential transcripts in Y1 and Y2 under low temperature stress for 0 day, 10 days, 20 days and 30 days. The x-axis shows differential transcript groups, and the y-axis shows the number of transcripts in these groups. **B** Venn diagram of different transcripts in Y1 and Y2 at different time points after low-temperature stress. GO functional enrichment analysis of differential transcripts in Y1 and Y2 under low-temperature stress for 10 days (**C**) and 20 days (**D**). KEGG functional enrichment analysis of differential transcripts in Y1 and Y2 under low-temperature stress for 10 days (**E**) and 20 days (**F**). The x-axis displays the number of transcripts belonging to the GO/KEGG function, and Y axis indicates the annotated GO/KEGG function

Differences in Y1 and Y2 at different stages of low-temperature induction were analyzed (Fig. 4A). It was found that the number of differential transcripts at 10 days and 20 days after low-temperature induction was 1379 and 1401, respectively. Interestingly, there were 367 and 187 up-regulated differential transcripts at 10 days and 30 days after low-temperature induction, respectively, which were more than down-regulated differential transcripts. However, there was little difference between them at other stages of low-temperature induction, suggesting that *I. indigotica* shows the strongest sensitivity at 10 days after vernalization. As shown in the Venn diagram (Fig. 4B), statistics of differential transcripts at different stages illustrated that there were 105 differential transcripts at 10 days and 108 differential transcripts shared at 20 days and 30 days, respectively, and the number of transcripts shared at 10, 20 and 30 days was 29, which represented that the 29 transcripts were continuously expressed during vernalization.

Attention was paid on 10 days and 20 days, when the number of differential transcripts was the largest, and GO enrichment was carried out for the differential transcripts at 10 days (Fig. 4C). The results manifested that the main enriched terms were hormone-related functions, including hormone response (GO: 0009725) and hormone-mediated signal transduction (GO: 0009755), gibberellin (GO: 0009739) and jasmonic acid (GO: 0009753), as well as flavonoids (GO: 0009813), carbohydrate metabolism (GO: 0044262) and active oxygen biosynthesis (GO: 1903409). At 20 days after low-temperature induction, chloroplasts (GO: 0009507), photosynthesis (GO: 0009521) and carbohydrate response (GO: 0009743), as well as hormone response (GO: 0009725), defense response (GO: 0006952) and low-temperature response (GO: 0009409) were found, most of which were associated with the stress response (Fig. 4D). Additionally, KEGG pathway enrichment analysis (Fig. 4E) was carried out, and the two pathways, namely, of zeatin biosynthesis (ko00908) and plant hormone signal transduction (ko04075) related to hormones, and secondary metabolite pathways such as phenylpropane metabolism (ko00940), fatty acid metabolism (ko00071) and flavonoid metabolism (ko00944) were mainly enriched at 10 days. Meanwhile, photosynthesis (ko00195) and MAPK signaling pathway (ko04016) were also enriched. At 20 days after low-temperature induction, the enriched pathways mainly included auxin-related tryptophan metabolism pathway (ko00380) and fatty acid (ko00062), folic acid (ko00790) and terpenoid (ko00905) secondary metabolism pathways. Besides, the photosynthesis (ko00195) pathway was also greatly affected. The above functions are crucial for plant growth and low-temperature stress.

### Construction of a co-expression network module

**Fig. 5:**
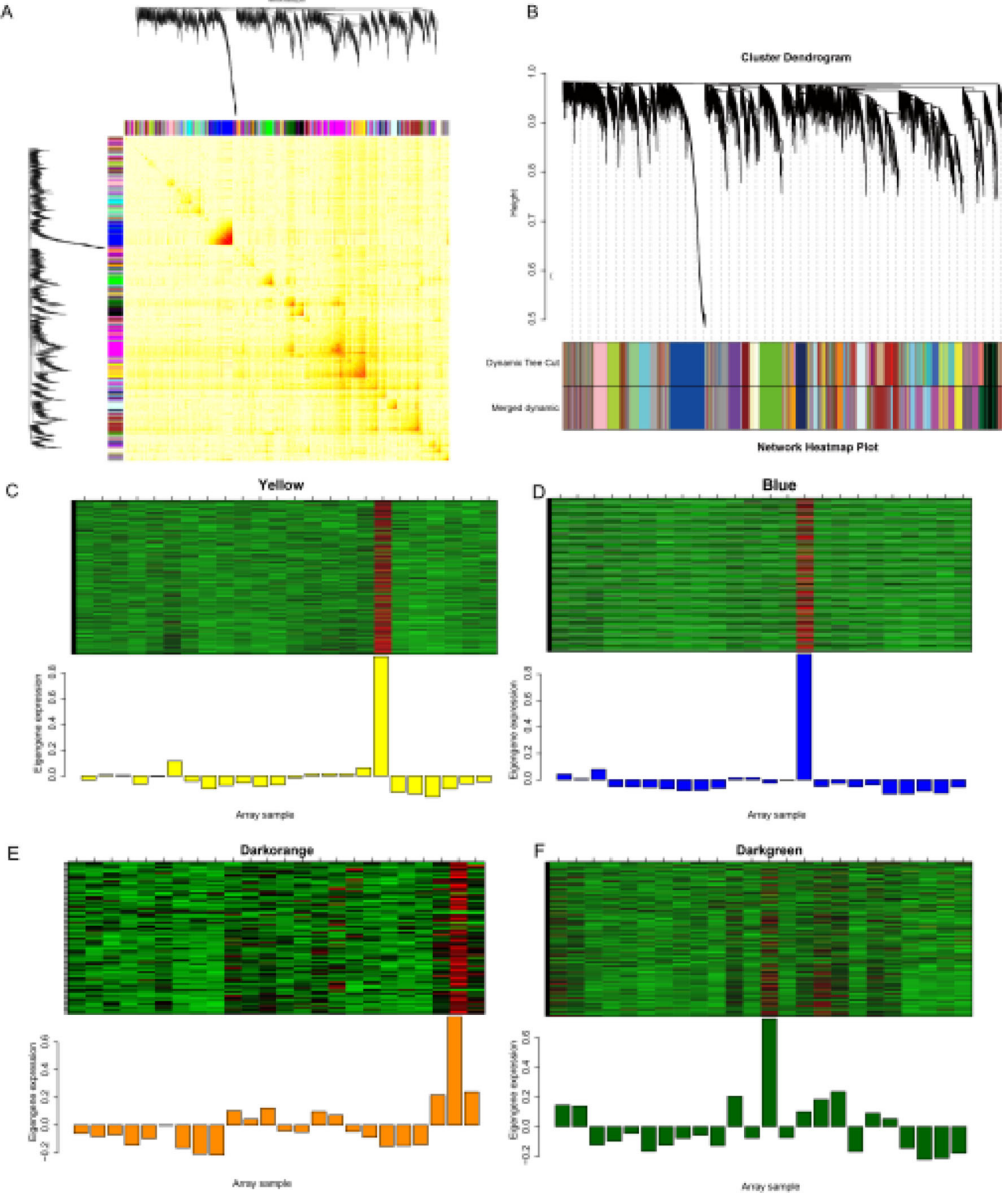
Co-expression network analysis of transcript genes in 24 T1 and T2 samples under low-temperature stress. **A** TOM heat map of correlations among all transcripts. **B** Co-expression network modules constructed by transcripts. Different colors represent the constructed network modules, the phylogenetic tree represents the hierarchical clustering of different samples, and each sample corresponds to one color of the modules. **C** Expression profile of transcripts in the co-expression Yellow (**C**), Blue (**D**), Darkorange (**E**), Darkgreen module (**F**). The x-axis of the histogram represents all modules, and the y-axis represents the expression profile of transcripts in all modules

To identify the key transcripts of *I. indigotica* in the process of low-temperature vernalization, a whole transcript co-expression network was constructed using 24 samples with a total of 15,410 transcripts, and the screening of transcripts with an expression level greater than 1 was performed to construct the co-expression network. A total of 25 co-expression clustering modules (Fig. 5B) were obtained by hierarchical clustering of dissTOM matrix (Fig. 5A). 4 modules were selected to show the transcript expression profile constructed by the co-expression network (Fig. 5C), and Yellow, Blue, Darkorange and Darkgreen modules displayed the transcript expression level under the co-expression clustering module.

### Identification of co-expression genes of endogenous metabolites of *I. indigotica* under low-temperature treatment

**Fig. 6:**
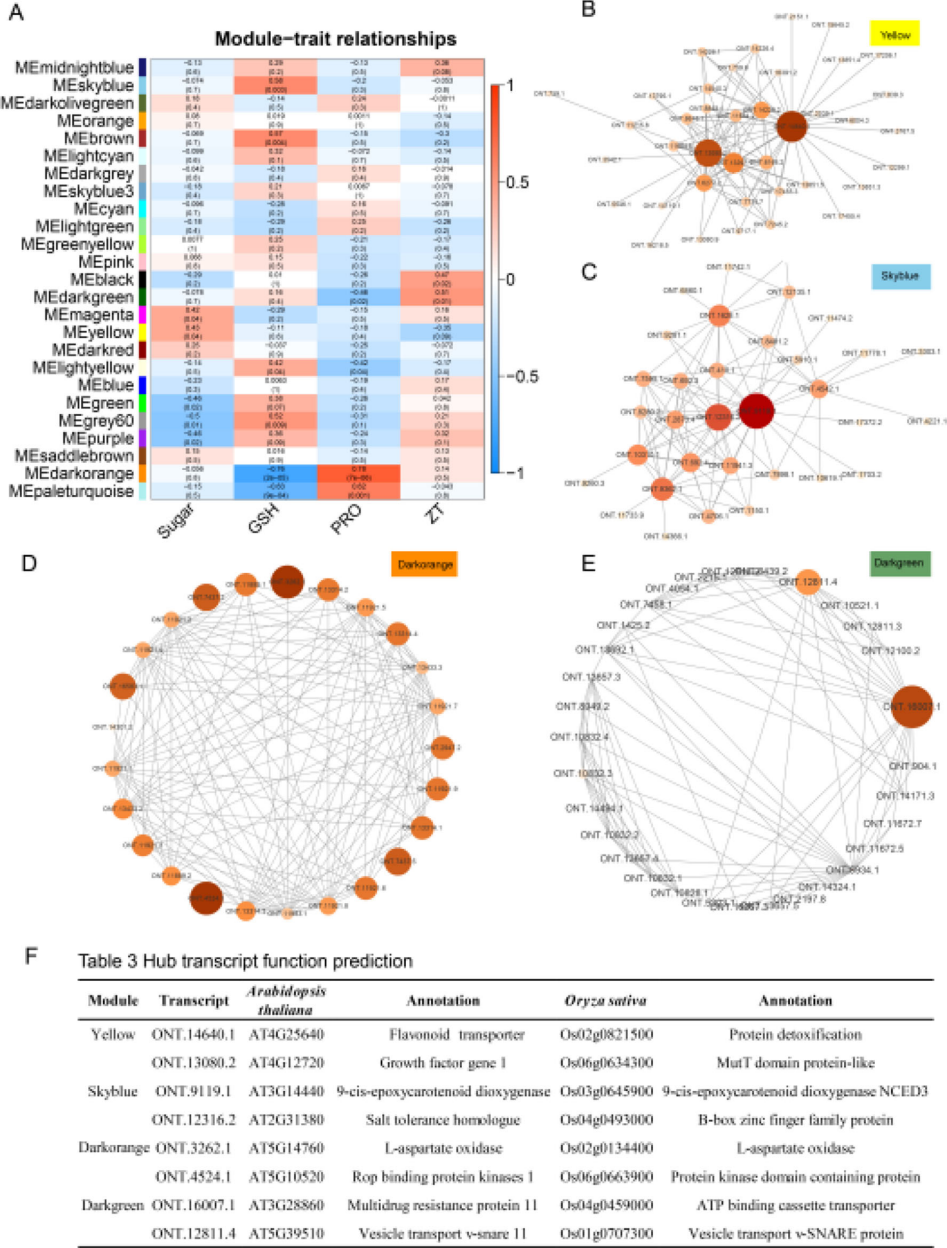
Core genes related to the concentrations of soluble sugar, glutathione, proline and zeatin in *I. indigotica* identified by co-expression modules. **A** Correlation heat map of the concentrations of soluble sugar, glutathione, proline and zeatin in *I. indigotica* identified by co-expression modules. In the x-axis, Sugar represents soluble sugar, GSH represents glutathione, PRO represents proline and ZT represents zeatin. A transcript co-expression network of identified modules related to soluble sugar (**B**), glutathione (**C**), proline (**D**), zeatin (**E**). **F** Functional prediction of the candidate hub gene

The correlations of the co-expression network with soluble sugar, glutathione, proline and zeatin were analyzed. Yellow module had the most significant correlation with soluble sugar (*r* = 0.43, *p* = 0.04), Skyblue exhibited the closest correlation with glutathione (*r* = 0.58, *p* = 0.0003), Darkorange exhibited the most obvious correlation with proline (*r* = 0.78, *p* = 7e−06), and Darkgreen displayed a close correlation with zeatin (*r* = 0.51, *p* = 0.01) (Fig. 6A). Subsequently, the key genes of modules showing close correlations with soluble sugar, glutathione, proline and zeatin were identified, and the module network was visualized using Cyttoscape. The core transcripts were *ONT.14640.1* and *ONT.13080.2* in Yellow module (Fig. 6B), *ONT.9119.1* and *ONT.12316.2* in Skyblue module (Fig. 6C), *ONT.3262.1* and *ONT.4524.1* in Darkorange module (Fig. 6D), and *ONT.16007.1* and *ONT.12811.4* in Darkgreen module (Fig. 6E). The functions of transcripts identified in the modules in *A. thaliana* and rice were predicted, respectively (Fig. 6F). In Yellow module, *ONT.14640.1* was annotated as a flavonoid transporter in *A. thaliana* and a detoxification protein in rice, *ONT.13080.2* was predicted as a growth factor in *A. thaliana* and a MutT domain protein in rice. In Skyblue module, *ONT.9119.1* was annotated as 9-cis-epoxycarotenoid dioxygenase (NCED3) in both *A. thaliana* and rice, and *ONT.12316.2* was annotated as a salt-tolerant homologue and a B-box zinc finger family protein. In Darkorange module, *ONT.3262.1* was annotated as L-aspartate oxidase in both *A. thaliana* and rice, and *ONT.4524.1* was annotated as ROP binding protein kinases 1 and protein kinase domain-containing protein. In Darkgreen module, *ONT.16007.1* was annotated as a multidrug resistance protein 11 in *A. thaliana* and ATP binding cassette transporter in rice respectively, and *ONT.12811.4* was annotated as Vesicle transport v-SNARE 11 in both *A. thaliana* and rice.

**Fig. 7:**
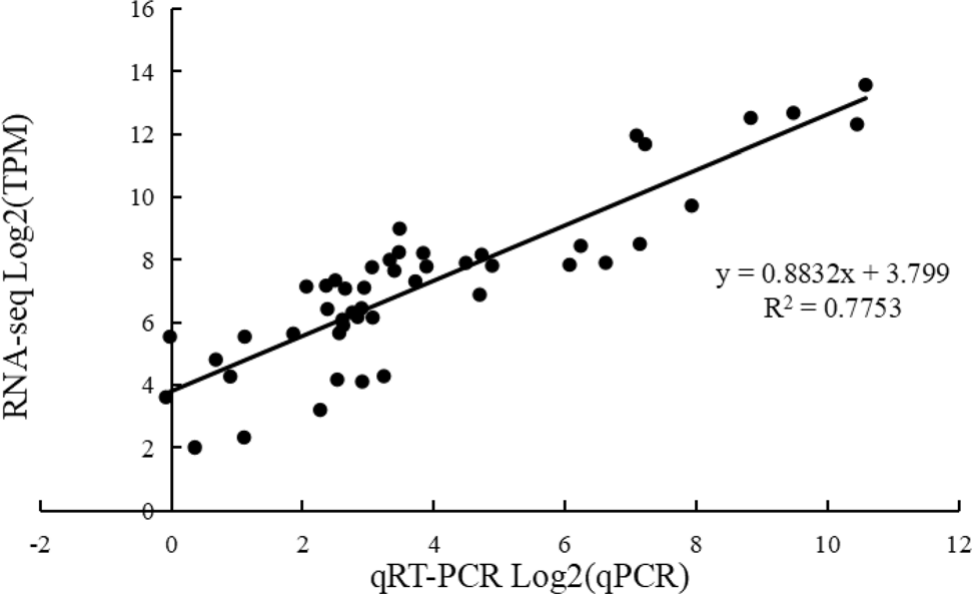
Comparison of RNA-seq and qPCR data of differentially expressed genes transformed using the log2fold-change (*r*^*2*^ = 0.78)

Later, with the aim of verifying the reliability of the transcriptome data, eight candidate transcripts, namely, *ONT.13080.2*, *ONT.13297.1*, *ONT.14640.1*, *ONT.17213.1*, *ONT.15448.1*, *ONT.12090.1*, *ONT.11185.1* and *ONT.16583.1*, were selected for quantitative real-time polymerase chain reaction (qRT-PCR) verification. Data obtained by transcriptome sequencing and qRT-PCR of these candidate transcripts were transformed by log2, after which the expression trends were compared between the two technologies. It was found that the expression trends were basically the same (*r*^*2*^ = 0.78; Fig. 7), which proved the reliability of the RNA-seq results.

## Discussion

*Isatis indigotica* is a crucial medicinal resource, but it is easily subjected to bolting and flowering after vernalization at low temperature, which induces the lignification in roots, thereby leading to the loss of its medicinal value. Therefore, clarifying the mechanism of *I. indigotica* in response to vernalization is highly valuable. In this study, the soluble sugar, glutathione, proline and zeatin in the two *I. Indigotica* germplasms, Y1 and Y2, were detected under continuous low-temperature induction. It was found that the concentration of glutathione was higher in Y1 than that in Y2, and the concentrations of soluble sugar, proline and zeatin were also higher in Y1 than those in Y2 at different stages. This is consistent with the results of low temperature stress in peach and kiwifruit. A large amount of proline accumulated in early stage of low temperature induction (Qian et al. 2021; Sun et al. 2021). Soluble sugar and proline, the main osmotic adjustment substances, are widely distributed in plants, which are related to the response of plants to low-temperature stress and will accumulate in plants to a certain extent under stress (Couée et al. 2006; Santarius 1973). Besides, the results manifested that soluble sugar and zeatin began to accumulate in Y2 at 10 days after low-temperature treatment, and the zeatin concentration in Y2 was dramatically higher than that in Y1. Zeatin is a natural cytokinin in plants crucial for the early abiotic stress of plants. It accumulates remarkably at 4 days after low-temperature induction in pea seedlings (Vaseva et al. 2009).

Nanopore sequencing is superior in the evaluation of the expression level of transcripts (Seki et al. 2019). Much more information can be provided by transcripts than by genes in the genome. For example, about 25,000 genes have 300,000 transcripts in *A. thaliana* (Hilson et al. 2004). In the present study, the vernalization process of *I. Indigotica* at low temperature was tracked in detail by Nanopore FL sequencing. After that, a total of 34,329 transcripts of *I. indigotica* were captured, 18,385 new transcripts were identified, and 11,169 CDS sequences, 6168 TFs and 30,952 SSR types were completely identified, which greatly enriched the data source of *I. indigotica*. LncRNAs are non-coding RNAs with 200 nt in length, and they have been reported to exert vital effects in plant growth and flowering (Zhao et al. 2018), reproductive development (Wang et al. 2014) and adversity stress (Cui et al. 2017). *VAS* has been identified as a lncRNA in wheat that can be induced to regulate the transcription of target gene *TaVRN1* and promote the flowering of wheat at the early stage of vernalization (Xu et al. 2021). In *A. thaliana*, lncRNA *SVALKA* related to cold induction has also been identified to be involved in the cascade regulation of CBF (Kindgren et al. 2019). In this study, 470 lncRNAs related to low-temperature induction were identified in total, and these newly identified lncRNAs laid a foundation for the research on the response of *I. indigotica* to vernalization at low temperature.

FL transcriptomes are able to identify key transcripts indispensable to regulation, which strongly supported the functional role. In this study, the whole transcript co-expression network of *I. indigotica* was constructed at low temperature using the identified transcripts. 25 co-expression modules were constructed using 15,410 transcripts filtered from 24 samples, and these modules were correlated with soluble sugar, glutathione, proline and zeatin receiving low-temperature induction of *I. indigotica*, and finally 8 related transcripts were identified. Because these transcripts were induced by low temperature stress of *I. indigotica*, the highly related transcripts identified after associating with these low temperature marker metabolite scan reflect the potential regulatory mechanism between genes and metabolites to a certain extent. Among them, *ONT.14640.1* identified in Yellow module was annotated as a flavonoid transporter in *A. thaliana.* Flavonoids exert crucial effects in plant growth and abiotic stress (Taylor and Grotewold 2005). The high concentration of flavonoids in apple varieties has been regarded as an important indicator for high storability and freshness retaining (Lu et al. 2021), suggesting that flavonoids play a vital role in the low-temperature vernalization of *I. indigotica*. Moreover, NCED3 was also identified in this study. NCED3, as a key enzyme for abscisic acid accumulation, encodes 9-cis-epoxycarotenoid dioxygenase 3, and it is widely reported that abscisic acid is involved in the low-temperature stress of plants (Sato et al. 2018). Abscisic acid, as an important plant hormone, is widely involved in plant reproductive development and stem (Liu et al. 2018; Xu et al. 2019). *ONT. 16,007.1* is highly homologous to *At3G28860* that can encode auxin transport facilitators in *A. thaliana*. The cold stress inducing changes in the plant growth and development is tightly linked to the intracellular auxin gradient (Rodrigues et al. 2009; Rahman 2013). Glutathione (GSH) is one of the most important factors for rice plants to resist cold stress, and a large amount of NAD + is required in this process (Yu et al. 2020). *ONT.3262.1*encodes l-aspartate oxidase in *I. indigotica*, and ATASPO is a key metabolic step to catalyze NAD^ +^ synthesis (Hao et al. 2018). The analysis that *ONT.3262.1* may regulate GSH accumulation in *I. indigotica* and needs to be verified by subsequent experiments. The above results reveal the reliability of WGCNA analyses in identifying key genes of *I. indigotica* at low temperature in this study. The low temperature stress of *I. indigotica* is a great significance for its delayed vermalization. In this study, the FL transcriptome sequencing technology was used to compare *I. indigotica* with different low temperature stress, and the transcripts related to low temperature stress were identified, including the functions involved in the synthesis, transport and metabolism of adscisic acid, auxin and flavonoids. It provides a certain research basis for molecular breeding of *I. indigotica* for better low temperature stress responses.

## Conclusions

*Isatis indigotica* is a vital medicinal plant resource, so analyzing its vernalization mechanism and exploring the genes resistant to low temperature are of great significance. In the current study, two transcripts of *I. indigotica* with different degrees of low-temperature tolerance were identified using Nanopore technology, and the important transcript types related to physiological indexes such as soluble sugar, glutathione, proline and zeatin were identified by WGCNA. The experimental results of this study strongly support the research on the vernalization mechanism of *I. indigotica* and the exploration of the genes resistant to low temperature.

## Supplementary Information

Below is the link to the electronic supplementary material.

Supplementary file1 (XLS 21 kb)

## Data Availability

Not applicable.

## Abbreviations

*I. indigotica:*: *Isatis indigotica* Fort.
*A. thaliana:*: *Arabidopsis thaliana*
SS: Soluble sugar
GSH: Glutathione
PRO: Proline
ZT: Zeatin
WGCNA: Weighted Gene Co-Expression Network Analysis
TFs: Transcription factors
FL: Full-length
ORFs: Open reading frames
CDSs: Complete coding sequences
COGs: Clusters of Orthologous Groups
KEGG: Kyoto Encyclopedia of Genes and Genomes
GO: Gene Ontology
NR: Non-Redundant Protein Sequence
CPC: Coding Potential Calculator
CNCI: Coding-Non-Coding Index
CPAT: Coding-Potential Assessment Tool
